# Implementing a benchmarking and feedback concept decreases postoperative pain after total knee arthroplasty: A prospective study including 256 patients

**DOI:** 10.1038/srep38218

**Published:** 2016-12-05

**Authors:** A. Benditz, J. Drescher, F. Greimel, F. Zeman, J. Grifka, W. Meißner, F. Völlner

**Affiliations:** 1 Department of Orthopedics, University Medical Center Regensburg, Asklepios Klinikum Bad Abbach, Kaiser-Karl-V-Allee 3, 93077 Bad Abbach, Germany; 2 Clinic for Anesthesia, Asklepios Klinikum Bad Abbach, Kaiser-Karl-V-Allee 3, 93077 Bad Abbach, Germany; 3 Centre for Clinical Studies, University Medical Center Regensburg, Franz-Josef-Strauss-Allee 11, 93053 Regensburg, Germany; 4 Department of Anesthesiology and Intensive Care, Jena University Hospital, Erlanger Allee 101, 07747 Jena, Germany

## Abstract

Perioperative pain reduction, particularly during the first two days, is highly important for patients after total knee arthroplasty (TKA). Problems are not only caused by medical issues but by organization and hospital structure. The present study shows how the quality of pain management can be increased by implementing a standardized pain concept and simple, consistent benchmarking. All patients included into the study had undergone total knee arthroplasty. Outcome parameters were analyzed by means of a questionnaire on the first postoperative day. A multidisciplinary team implemented a regular procedure of data analyzes and external benchmarking by participating in a nationwide quality improvement project. At the beginning of the study, our hospital ranked 16^th^ in terms of activity-related pain and 9^th^ in patient satisfaction among 47 anonymized hospitals participating in the benchmarking project. At the end of the study, we had improved to 1^st^ activity-related pain and to 2^nd^ in patient satisfaction. Although benchmarking started and finished with the same standardized pain management concept, results were initially pure. Beside pharmacological treatment, interdisciplinary teamwork and benchmarking with direct feedback mechanisms are also very important for decreasing postoperative pain and for increasing patient satisfaction after TKA.

The 40 surgical procedures with the highest pain scores (median Numerical Rating Scale (NRS) 6 or 7) include 22 orthopedic interventions on the extremities. Total knee arthroplasty (TKA) is in 5^th^ place of these TOP 40 surgical procedures. In addition, a major factor for patient dissatisfaction, prolonged hospital stays with delayed recovery, and immobility after surgery is severe postoperative pain^1^ which may be associated with the development of chronic pain. Total knee replacement is highly stressful for patients. After total knee arthroplasty (TKA), 75.0% of patients complain about moderate to severe pain^2^. The worst mean postoperative pain score on the NRS in 1,247 analyzed patients was 5.56 (±2.66), showing a high correlation with the development of chronic pain^3^.

To improve postoperative pain and patient satisfaction, many studies tried to optimize techniques and drugs^4^,^5^. In general, nerve blockage is superior to patient-controlled analgesia (PCA) in TKA^2^,^6^,^7^,^8^,^9^. Despite heightened awareness and clinical advancements in pain management, the quality of acute postoperative pain management is still far from satisfactory as shown by Gan in 2014^10^.

Therefore, it is very important to find new ways to improve postoperative pain management. In a present study Chughtai showed that patients are of the opinion that postoperative pain control after TKA is mainly the responsibility of the treating orthopedist^11^. As we believe that inadequate pain treatment is often caused by organizational problems and lack of sufficient staff education in pain issues^12^,^13^, not only the physician but the entire healthcare team should be responsible for pain management. Particularly nurses who have much more contact with patients are important to be well trained in pain management^14^,^15^. Education and awareness are a pre-requisition for detecting deficits and implementing evidence-based pain management strategies. Moreover, proper training of staff might improve communication and empathy for the pain patient. Regarding the literature there are positive effects described by standardized data acquisition and an analysis of quality and process indicators^16^. But for orthopedic procedures such as TKA, there are no findings in the literature that the quality of postoperative pain management may be improved by implementing continuous quality improvement (CQI) strategies to detect and adjust insufficient pain management. Key elements of CQI include continuous re-assessment and analysis of processes and outcomes^17^.

Our hypothesis was that pain management can also be improved by continuous benchmarking and implementation of a pain management concept including feedback from and to educated staff. Such improvements subsequently reduce postoperative pain and improve patient satisfaction. Our specific pain management concept was agreed on by an interdisciplinary pain council. Although postoperative pain is multifactorial, this study was not aimed at discussing the concept itself but at finding out whether the manner in which the concept is implemented by the medical team may make a difference.

## Methods

### Data assessment

The present prospective cohort study included the registry data of 256 patients who had undergone total knee replacement surgery at our university medical center between 2014 and 2015. The data were collected for the project ‘Quality Improvement in Postoperative Pain Treatment’ (QUIPS), a benchmark initiative for comparing pain outcome parameters among participating hospitals. This project has been supported by the German Society of Anesthesiologists and the German Society of Surgeons^16^,^18^. Patients older than 18 years who had received primary TKA and were able to communicate were included. Exclusion criteria were: (1) patients not present at the ward at the time of data collection; (2) patients who had visitors at the time of data collection; (3) patients who refused to participate in the study; (4) patients who were sedated or asleep or had cognitive dysfunction. Process data including pre-operative, intra-operative, and postoperative variables were collected from the medical records on postoperative day 1.

The validated 15-item questionnaire asked for worst and least pain intensities since surgery using a numeric rating scale (NRS: 0 = no pain, 10 = worst pain imaginable)^19^. A specialized pain nurse visited a random sample of patients on the first postoperative day. Wards to be visited were randomized daily to prevent selection bias. The nurse interviewed and documented postoperative pain treatment as well analgesia-related complications. To avoid any interviewer-patient interaction bias, the nurse informed the patients that she was working independently from the healthcare team, that all information or judgements given in the interview would be treated confidentially, and that participation was voluntary. Data were anonymized after the interview. The primary endpoint of the study was a change in NRS for pain and satisfaction.

### Ethical approval and informed consent

The project was approved by the Ethics Committee as well as the Data Security Board of the Jena University Hospital, Jena, Germany, as well as by the Ethics Committee of the University of Regensburg. The study is registered in the German Register of Clinical Studies (DRKS) under the number DRKS00006153 (WHO register). The study was carried out in accordance with the ethical standards of the Declaration of Helsinki of 1975. Patients were informed in written form as well as orally by the study personnel. An informed consent was obtained from all subjects. Participation was voluntary, and withdrawal was possible at any time.

### Pain management concept

Our standard pain management concept for patients undergoing TKA was used for each patient in this study.

1 h before surgery, patients receive oral benzodiazepine premedication followed by ischiadic nerve and psoas compartment blockage with 20 ml of ropivacaine 0.75% and 20 ml of prilocaine 1.0% for each block. Patients are sedated with propofol during surgery. During the first 12 h after surgery, the nurse in the intermediate care unit administers 3 mg of piritramid on demand in intervals. 0.375% of ropivacaine set at 6–10 ml/h is used for nerve blockage and Ibuprofen 600 (3×) as standard analgesic on a regular daily basis. At the normal ward, oral controlled analgesia (OCA) is administered. Depending on the NRS, patients may get additional analgesics if required: Tramadol 100 mg (40gtt) with the possibility of a repeated dose after 30 min if NRS 3–6 and oxycodone 20 mg and a repeated dose after 1 h if NRS 7–10. In the case of persisting or increasing pain, the nurse will notify the physician. In addition, patients are advised on how to avoid pain by self-activation and are asked to report any occurrence of pain as well as its characteristics, also during night time. Cool packs for the affected knee are also provided.

### Benchmarking and feedback

A multidisciplinary team of anesthetists, orthopedic surgeons, and nurses implemented a regular procedure of data analyzing and internal benchmarking. Beside the main parameters mean NRS max. pain, min. pain, activity-related pain and patient satisfaction, side effects like nausea, dizziness, tiredness and postoperative nausea and vomiting (PONV) prophylaxis were evaluated. In addition functional parameters were analysed, that means patients were asked how much pain affected their ability to move in bed, their ability to cough or deep breath, their ability to sleep and their mood in the last 24 h hours after surgery. The healthcare team was informed on any results and suggested improvements. Every staff member involved in pain management had educational lessons every 3 months, and a special pain nurse was trained in each ward. The data shown here were internally analyzed for each ward to be able to give a direct feedback to the ward of the respective patient. Additional lessons were given in the case of deteriorating standards or a turnover in staff because of ward restructuring.

Nurses received lessons in general pain management, in pain treatment required by patients after TKA, and pharmacological training according to our standards in pain management. Nurses were encouraged to use all treatment options available and were informed about possible risks. In addition, we emphasized the importance of using non-pharmacological therapeutic possibilities such as cooling the wound and different positioning of the patients as well as of communication with the patient and between nurse and physician.

Additionally, patients were asked to report pain to the nurses as early as possible and not try to bear the pain.

### Statistical methods

Statistical analysis was done with SPSS (IBM SPSS Statistics, Version 23.0., Armonk, NY: IBM Corp.). All single results were divided into intervals of 3 months (quarter of a year) to establish a timeline. Metric variables were reported descriptively as mean and standard deviation. Statistical data were not normally distributed. Non-parametric Mann-Whitney U tests were used to compare continuous variables between resulting independent subgroup pairs and the Kruskal-Wallis test to compare results between multiple subgroups. Pearson’s Chi-square tests were used to compare categorized data of independent subgroups. Analyses included the chi square test and the non-parametric Mann-Whitney U test to compare the effects. Statistical significance was set at a level of P < 0.05. With a sample size of n = 112 in 2014 compared to n = 144 in 2015, we had 80% power to detect an effect size of d = 0.35, which can be considered as small.

## Results

### General results

256 patients who had undergone primary total knee arthroplasty surgery between January 2014 and December 2015 were included in this study. The mean age was 66.87 years (±10.00), and 89.1% of the patients had an ASA (American Society of Anesthesiologists Physical Status classification system) status of 2 or 3. Surgery was conducted by experienced orthopedic surgeons at a center of excellence for arthroplasty (Department of Orthopedics of the University of Regensburg). The mean duration of surgery was 81.65 min (±24.16). 72.7% of patients had reported chronic pain in the affected knee for more than 3 months prior to surgery with a mean NRS of 6.95 (±1.65). (Table 1) 82% of the patients took peripheral analgesics, e.g. ibuprofen on a regular daily base. 34% of those also took opioid analgesics.

### Side effects and functional parameters

A comparison of the side effects between 2014 and 2015 showed improvement in all parameters. Nausea, tiredness, and dizziness just showed a tendency toward decrease, but other functional influences were significantly decreased. The decrease in the interference of pain with the ability to move had significantly dropped from 60.5% to 29.5% (p = 0.001). In 2014, 14.7% of patients had reported their mood being influenced by pain in contrast to only 1.2% in 2015 (p < 0.001). The number of patients receiving nausea prophylaxis (PONV) had also risen from 30.9% in 2014 to 35.4% in 2015. (Table 2) Number of patients reporting nausea showed an inconsistent pattern.

### Pain

**Figure 1:**
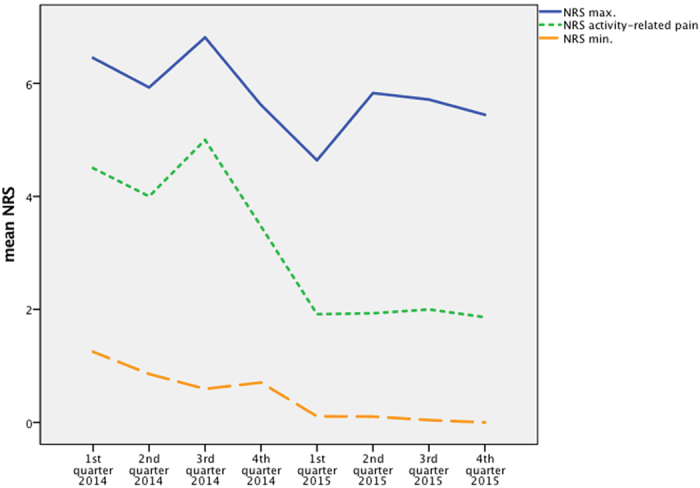
Timeline of mean NRS maximum, minimum and activity-related pain.

Mean NRS (0–10) were recorded for maximum pain, minimum pain, and activity-related pain (e.g. movement). At the beginning, mean maximum pain was 6.45 (±2.235), which could be decreased to 5.93 (±2.56) in the 2^nd^ quarter of 2014. This score rose to 6.81 (±2.24) in the 3^rd^ quarter before continuously falling to the minimum of 4.64 (±1.88) in the 1^st^ quarter of 2015. There was a massive rise to 5.83 (±1.39) before it could be reduced again to 5.44 (±1.83) at the end of the observation period (p = 0.001) (Fig. 1).

Minimum pain started at a mean NRS of 1.25 (±1.37) and nearly permanently decreased to a value of 0 (±0) over the last two quarters of 2015, which represented significant improvement (p < 0.001) (Fig. 1).

Activity-related pain overall followed the curve of maximum pain. Starting at a mean of 4.50 (±2.50), a first decrease was followed by an increase to 5.00 (±2.45). In the 1^st^ quarter of 2015, the score dropped to 1.91 (±1.23) and remained constant at 1.86 (±1.07) until the end of the surveillance period. This improvement was also significant (p < 0.001) (Fig. 1).

After implementation of the program, an (non-significant) increase in the use of piritramid and tramadol could be noticed.

**Figure 2:**
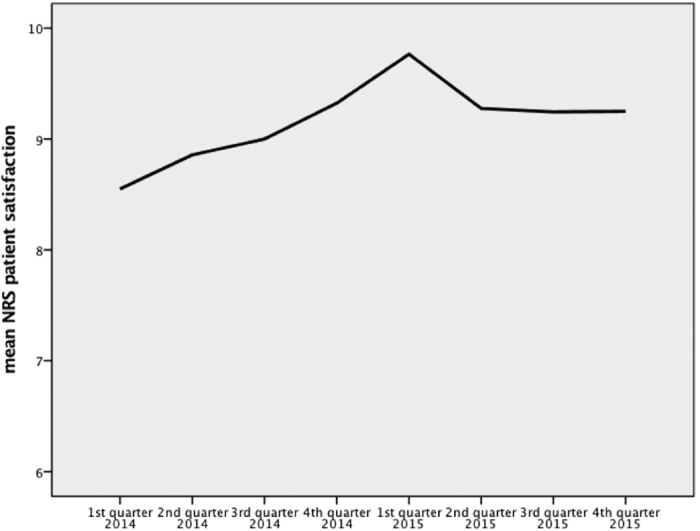
Timeline of mean NRS patient satisfaction.

Patient satisfaction was also recorded on a NRS. At the beginning, patient satisfaction was 8.55 (±1.54), rising continuously to 9.77 (±0.56) in the 1^st^ quarter of 2015 that was followed by a small bend. After that, the patient satisfaction score remained nearly constant at 1.86 (±1.07) until the end of the study (p = 0.001) (Fig. 2).

### Comparison among 47 anonymized hospitals

**Figure 3:**
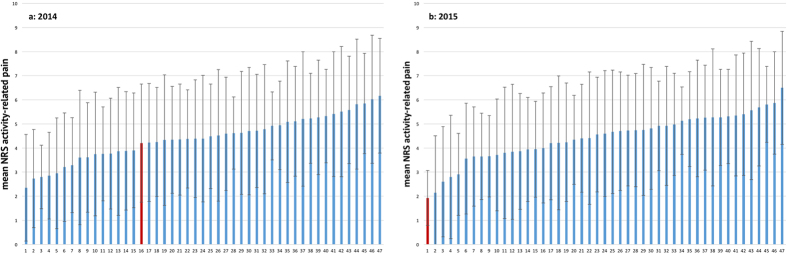
(**a**,**b**) Comparison of mean NRS activity-related pain among 47 anonymized hospitals in 2014 (**a**) and 2015 (**b**). The red bar shows our hospital.

**Figure 4:**
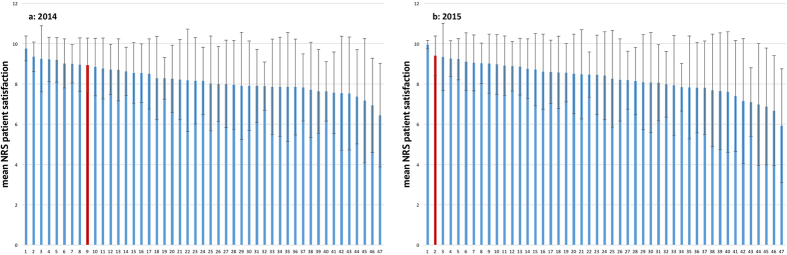
(**a**,**b**) Comparison of mean NRS patient satisfaction among 47 anonymized hospitals in 2014 (**a**) and 2015 (**b**). The red bar shows our hospital.

In comparison to the other 46 hospitals, our clinic started at rank 16 for activity-related pain (Fig. 3a) and at rank 9 for patient satisfaction (Fig. 4a) in 2014. In 2015, we had improved to 1^st^ place in activity-related pain (Fig. 3b) and to 2^nd^ place in patient satisfaction (Fig. 4b).

## Discussion

In this study we wanted to show that pain management can be improved by consistent benchmarking and implementation of a pain management concept including feedback from and to educated staff and that such improvements subsequently reduce postoperative pain and improve patient satisfaction.

Over the past years, pharmacological standards and surgical methods − both highly influential factors on postoperative pain − have seen many improvements. Although postoperative pain is multifactorial, we focused on benchmarking in a standardized setting.

Even in 1994 Rawal stated: ‘However, it is increasingly recognized that the solution to the problem of inadequate pain relief on surgical wards lies not so much in the development of new drugs and new techniques but in the development of a formal organization for better use of existing drugs and techniques’^13^. The lack of interdisciplinary commitment, concerns, poor reimbursement for interdisciplinary care, and the lack of staff are often the cause of this complication^20^.

Against this background, we started to actively participate in our multidisciplinary pain council, also in view of the recertification that was due at the end of 2014 (©Paincert). After a first period of collecting data, we started at rank 16 for activity-related pain and at rank 9 for patient satisfaction. Maximum pain was 6.45 (±2.235) and minimum pain 1.25 (±1.37). In another recent study Gerbershagen showed a mean max. pain of 5.56 (±2.66) postoperatively^1^. After analyzing the data, intensive training sessions for healthcare providers were started. Both physicians and nurses received lessons in general pain management, in pain treatment required by patients after TKA, as well as pharmacological training according to our standards in pain management. The participants were encouraged to use all treatment options available and were informed about possible risks. In addition, we emphasized the importance of using non-pharmacological therapeutic possibilities such as cooling the wound and different positioning of the patients. Recent studies have shown that ‘perception of orthopedists and nurses both outweigh perception of pain control on overall surgical experience. Orthopedists should focus on staff education − particularly nurses − and educate them in order to optimize results and, ultimately, improve patient satisfaction’^11^. In 2006 Meissner *et al*. also showed that a continuous quality improvement process could be established in clinical routine over three surgical departments. ´Cornerstones of that project were frequent assessments of process and outcome parameters, regular benchmarking and implementation of feedback mechanisms´^17^.

First positive results were seen rather soon, because the NRS for maximum and minimum pain had decreased at the end of the 2^nd^ quarter of 2014. Patient satisfaction had also increased immediately. Because of these positive results, staff training was set back to normal because the time needed for additional training had been difficult to integrate into clinical routine in the first 6 months. This decision was perceived as negative, and all parameters worsened over the next period of data collection. Because of this relapse and the impending recertification, the decision to cut down on staff training was revised, and training lessons were again intensified. Particularly newly appointed physicians and nurses got extra lessons to perform better within our pain management concept, which significantly decreased all pain scales as well as side-effects in 2015. In this year, the educational program was driven by staff demand, resulting in a satisfactory steady state. At the end of the study, our department ranked 1^st^ in activity-related pain and 2^nd^ in patient satisfaction in the inter-hospital comparison. These positive results were reached without increasing the negative side-effects significantly.

When comparing these data to other present studies, there are no similar findings in the field of orthopedics. But also using QUIPS, similar positive effects have been described in improving pain management after septorhinoplasty^21^ or comparing pain after caesarean section^18^.

Outside Germany, the corresponding project PAIN OUT (www.pain-out.eu) offers a similar tool for feedback and benchmarking.

The implementation of continuous benchmarking has been shown to help decrease postoperative pain in all types of surgical treatment. Collecting postoperative data may aid the recognition and solving of any existing problems. Even in countries without such a nationwide project, single hospitals or associated hospitals may implement benchmarking by comparing the data of different wards within the hospital. Wards or hospitals with lower marks could benefit from the experience of those with higher marks.

Obviously, the more difficult the situation is at the beginning, the better is the chance of improving. But our results have shown the difficulties in maintaining improvements over a long period^16^,^22^. When analyzing such benchmarking, the ‘Hawthorne effect’ has to be considered. Success is often not triggered by the process itself but is rather caused by the psychological stimulus of healthcare providers. As long as the process is ongoing, people get a certain amount of attention, but lose interest as soon as the project is finished^23^,^24^.

Despite the benefits of QUIPS, such as standardized data acquisition with validated questionnaires and trained nurses conducting the interviews, this study also has some limitations. We have no information about the excluded patients. Patients who were not present at the ward or did not want to participate are not represented in the registry, which may be a potential source of bias. Furthermore, postoperative pain and pain management were only assessed within the first 24 h, as the QUIPS protocol only compares these data to other hospitals. Although our own studies have also shown that pain peaks on the first postoperative day^25^,^26^, a longer follow-up should be planned for further studies. This would offer the possibility to observe functional parameters (e.g., mobilization) as well. In addition, patients were only interviewed Tuesdays to Fridays, because on weekends no pain nurse was available to collect data. As surgery was not conducted on Sundays, no patients were available on Mondays.

## Conclusion

This study showed successful improvements in postoperative pain management by establishing a CQI process that we have been using in clinical routine for over 2 years. All parameters of process and outcome quality improved in the first interval of the observation period and remained constant for over 1 year. Our results suggest that − next to standardized high-quality pharmacological treatment − interdisciplinary teamwork and benchmarking with direct feedback mechanisms might be also very important for decreasing postoperative pain and for increasing patient satisfaction after TKA. Yet, first improvements should not lead to complacency.

## Additional Information

**How to cite this article**: Benditz, A. *et al*. Implementing a benchmarking and feedback concept decreases postoperative pain after total knee arthroplasty: A prospective study including 256 patients. *Sci. Rep.* **6**, 38218; doi: 10.1038/srep38218 (2016).

**Publisher's note:** Springer Nature remains neutral with regard to jurisdictional claims in published maps and institutional affiliations.

## Figures and Tables

**Table 1:** Demographic data of the 256 patients included.

Parameter	Mean ± SD		Range	
Age (yr)	66.87 ± 10.00		25–85	
Sex (m:f)	101:153			
Duration of surgery (min)	80.31 ± 19.99		34–168	
ASA Score	1	2	3	4
Frequency (%)	10.5	59.4	29.7	0.4
Chronic pain >3 months before surgery				
in the operated region	186		81.9	
in the operated region and 1 other region	40		17.6	
NRS of chronic pain in the operated region	6.95 ± 1.65		3–10	
NRS of chronic pain over all	6.93 ± 1.69		3–10	

**Table 2:** Side-effects and functional parameters.

Side-effects since surgery	2014 (n = 112)	2015 (n = 144)
Nausea (%)	23.2	31.1
Dizziness (%)	30.5	22.4
Tiredness (%)	48.4	36.6
PONV prophylaxis (%)	30.9	35.4
Functional parameters since surgery		
Pain affected ability to move (%)	87.4	69.6^*^
Pain affected ability to cough or take a deep breath (%)	2.1	1.2
Pain affected ability to sleep (%)	29.5	37.3
Pain affected mood (%)	14.7	1.2*

^*^(p < 0.001).
